# Myco-Synergism Boosts Herbivory-Induced Maize Defense by Triggering Antioxidants and Phytohormone Signaling

**DOI:** 10.3389/fpls.2022.790504

**Published:** 2022-02-17

**Authors:** Raufa Batool, Muhammad Jawad Umer, Yangzhou Wang, Kanglai He, Muhammad Zeeshan Shabbir, Tiantao Zhang, Shuxiong Bai, Jie Chen, Zhenying Wang

**Affiliations:** ^1^State Key Laboratory for Biology of Plant Diseases and Insect Pests, Institute of Plant Protection, Chinese Academy of Agricultural Sciences, Beijing, China; ^2^State Key Laboratory of Cotton Biology, Institute of Cotton Research, Chinese Academy of Agricultural Sciences (ICR, CAAS), Anyang, China; ^3^Insect Ecology, Institute of Plant Protection, Jilin Academy of Agricultural Sciences, Changchun, China; ^4^Institute of Plant Protection, Guangdong Academy of Agricultural Sciences, Guangzhou, China; ^5^School of Agriculture and Biology, Shanghai Jiao Tong University, Shanghai, China

**Keywords:** myco-synergism, *Ostrinia furnacalis*, *Beauveria bassiana*, *Trichoderma asperellum*, antioxidants myco-synergism triggers herbivory-induced defense

## Abstract

**Background:**

Biocontrol strategies are the best possible and eco-friendly solution to develop resistance against *O furnacalis* and improve the maize yield. However, the knowledge about underlying molecular mechanisms, metabolic shifts, and hormonal signaling is limited.

**Methods:**

Here, we used an axenic and a consortium of entomopathogenic *Beauveria bassiana* OFDH1-5 and a pathogen-antagonistic *Trichoderma asperellum* GDFS1009 in maize and observed that consortium applications resulted in higher chlorophyll contents and antioxidants activities [superoxide dismutase (SOD), peroxidase (POD), proline, protease, and polyphenol oxidase (PPO)] with a decrease in *O. furnacalis* survival. We performed a comprehensive transcriptome and an untargeted metabolome profiling for the first time at a vegetative stage in fungal inoculated maize leaves at 0-, 12-, 24-, 48-, and 72-h post insect infestation.

**Results:**

The consortium of *B. bassiana* and *T. asperellum* leads to 80–95% of *O. furnacalis* mortality. A total of 13,156 differentially expressed genes were used for weighted gene coexpression network analysis. We identified the six significant modules containing thirteen candidate genes [protein kinase (GRMZM2G025459), acyl-CoA dehydrogenase (GRMZM5G864319), thioredoxin gene (GRMZM2G091481), glutathione *S*-transferase (GRMZM2G116273), patatin-like phospholipase gene (GRMZM2G154523), cytochrome P450 (GRMZM2G139874), protease inhibitor (GRMZM2G004466), (AC233926.1_FG002), chitinase (GRMZM2G453805), defensin (GRMZM2G392863), peroxidase (GRMZM2G144153), GDSL- like lipase (AC212068.4_FG005), and Beta-glucosidase (GRMZM2G031660)], which are not previously reported that are highly correlated with Jasmonic acid - Ethylene (JA-ET) signaling pathway and antioxidants. We detected a total of 130 negative and 491 positive metabolomic features using a ultrahigh-performance liquid chromatography ion trap time-of-flight mass spectrometry (UHPLC-QTOF-MS). Intramodular significance and real time-quantitative polymerase chain reaction (RT-qPCR) expressions showed that these genes are the true candidate genes. Consortium treated maize had higher jasmonic acid (JA), salicylic acid (SA), and ethylene (ET) levels.

**Conclusion:**

Our results provide insights into the genetics, biochemicals, and metabolic diversity and are useful for future biocontrol strategies against ACB attacks.

## Introduction

Maize plants undergo several challenges during their growth, including pathogenic infections, environmental stresses, and insect attacks (Batool et al., 2020). Asian corn borer, *Ostrinia furnacalis* (Guenée), is among the most destructive insect pests of maize in Southeast and East Asia, especially China, causing an estimated yield loss of 6–9 million tons annually (He et al., 2003). Based on the importance of sustainable and eco-friendly agricultural practices (Guo et al., 2019), *O. furnacalis* control strategies have been shifted toward the application of biological control agents rather than harmful chemical pesticides (Mitchell et al., 2016; Batool et al., 2020). The *Trichoderma* spp. is traditionally viewed as a kind of biocontrol fungi against a range of plant pathogens (Dou et al., 2020), however, *Trichoderma* spp. has also been found with some functions against plant insects in different ways. More interestingly, in our previous study, we proved that *T. asperellum* and *Beauveria bassiana* are great entomopathogenic against *O. furnacalis* in maize (Batool et al., 2020). The plant herbivory response is a dynamic process that temporally and spatially regulates proteins, metabolic profiles, and transcript patterns (Ehlting et al., 2008; Barah and Bones, 2015). This suggests that it is essential to study the plant herbivory defense mechanisms at transcriptional and metabolic levels in response to insect-feeding and pre-inoculated entomopathogenic fungal strains (Li et al., 2016).

To combat herbivory, plants have evolved a broad range of defense mechanisms (Abebe, 2021). The morphological structures such as trichomes, waxy leaf surface, and numerous secondary metabolites act as the first barrier or the constitutive defense against herbivore attack. Plants have also developed an induced defense response, which affects the herbivore’s survival, growth, and development directly through the production of defensive proteins and secondary metabolites, and indirectly through the secretion of volatile compounds to attract the pests (Guo et al., 2019). Plant-induced defense is initiated by the perception of insect-feeding-derived elicitor, and it is a sophisticated mechanism to balance the growth and defense. When plants are exposed to a herbivore attack, different receptors and transporters could be activated and pass the signals through different signal molecules to activate the genes involved in the biosynthesis of phytohormones such as jasmonic acid (JA), ethylene (ET), and salicylic acid (SA), secondary metabolites, and defensive enzymes, which are toxic, repellent, interfere in the digestive system, survival, and nutrient absorption of insects (Qi et al., 2018). JA and SA accumulate and exhibit a major role in regulating the plant defense mechanisms against herbivory (Tzin et al., 2015; Guo et al., 2019). JA-induced signaling is primarily regulated upon insect feeding, whereas salicylic acid regulates the response to phloem-feeding insects (Tzin et al., 2015; Heidel-Fischer et al., 2018). Herbivory-induced defensive enzymes, including enzymatic [superoxide dismutase (SOD), peroxidase (POD), polyphenol oxidase (PPO), and protease] and non-enzymatic (proline) antioxidants in maize plants, help to develop resistance against a corn borer and are responsible for the regulation of plant defense mechanism through the production of secondary metabolites and reactive oxygen species (ROS) scavenging (Batool et al., 2020). *Trichoderma* is also capable of stimulating an induced systemic resistance (ISR) plant defense response against aphids, caterpillars, and nematodes, modulated by crosstalk between SA and JA (Di Lelio et al., 2021).

Entomopathogenic fungi have traditionally been assumed to help regulate insect populations. So far, they have aroused a limited interest as plant growth promoters, alongside of protecting plant form herbivores (González-Guzmán et al., 2020, 2021). However, some hypocrealean ascomycetes, such as *B. bassiana*, play other poorly understood ecological roles that might be useful in developing novel strategies for both increased crop production and crop protection (Sánchez-Rodríguez et al., 2018). Sánchez-Rodríguez et al. (2018) concluded in their study that *B. bassiana* successfully colonized the plant and boosted the spike production in wheat inoculated with seed-dressing and soil drenching methods and caused a 30–75% larval mortality after consuming the endophytically-colonized leaves. In their work, González-Guzmán et al. (2020) also examined the performance of two wheat varieties inoculated with entomopathogenic fungi including *B. bassiana* and *Metarhizium brunneum* by soil drenching and seed coating method and found an increased yield with no significant difference in plant heights. Their results support the seed-dressing method of inoculating with entomopathogenic fungi as a promising green technology for crop protection and enhancement with no negative effect on plant performance. Landa et al. (2013) identified the endophytic colonization of the Opium poppy plant by *B. bassiana* and concluded that it can protect the plant from stem gall wasp. The *B. bassiana, M, brunneum*, and *Isaria farinosa* were also found to increase the iron (Fe) availability in soil differently depending on particle size, increased plant height, and inflorescences the production of sunflower grown (Raya-Díaz et al., 2017).

Advances in omics and quantitative biology offer several ways to identify gene networks and their regulatory mechanisms in living systems. One such promising approach is the RNA-Sequence-based weighted gene co-expression network analysis (WGCNA) (Langfelder and Horvath, 2008). The WGCNA is a useful method to identify modules/networks of coexpressed genes. Furthermore, the correlation of these modules with phenotypic traits was useful to detect the key genes within the networks (Umer et al., 2020). However, no such large-scale study regarding the synergistic interaction of *B. bassiana* OFDH1-5 and *T. asperellum* GDFS1009 in triggering the omics-based regulatory mechanisms of defense genes, biochemical enzymes, and phytohormones induced by the *O. furnacalis* herbivory, are available.

Here, we hypothesized that the consortium of entomopathogenic *B. bassiana* OFDH1-5 and pathogenic antagonistic *T. asperellum* GDFS1009 fungal inoculants can trigger the enhanced defense response in maize, then the normal herbivory-induced response by an enhanced expression of defense-related genes, antioxidants production, phytohormone synthesis, digestive enzyme inhibitors, etc. So, we performed the physiological, biochemical, transcriptome, metabolome, and phytohormonal level analysis in field-grown vegetative stage maize inoculated with *B. bassiana* OFDH1-5 and *T. asperellum* GDFS1009, singly and in the consortium at 0-, 12-, 24-, 48-, and 72-h under *O. furnacalis* feeding. By integrating differentially expressed genes (DEGs) and biochemical antioxidants through weighted gene coexpression network analysis (WGCNA), we revealed gene networks and key candidate genes linked to JA, SA, and ethylene biosynthesis pathway and production of antioxidants and insect cuticle and digestive enzyme inhibiters. Our results highlight the positive impact of *B. bassiana* OFDH1-5 and *T. asperellum* GDFS1009 on increasing the herbivory-induced defenses and restricting the survival and growth of *O. furnacalis*.

## Materials and Methods

### Plant Growth and Treatments

Maize variety “Jingke 968” was planted in an open field located at the Gongzhuling Experimental Station of the Institute of Plant Protection, CAAS, Gongzhuling, China (43°30′ N, 124°47′ E; 224.9 m above sea level) on May 11, 2018, and May 06, 2019. The treatments include: (CK) untreated control, (BB-1) seed treatment with *B. bassiana* OFDH1-5, (BB-2) soil drenching with *B. bassiana* OFDH1-5, (TH-1) seed treatment with *T. asperellum* GDFS1009, (TH-2) soil drenching with *T. asperellum* GDFS1009, (BT-1) seed treatment with consortium of *B. bassiana* OFDH1-5 and *T. asperellum* GDFS1009, (BT-2) soil drenching with a consortium of *B. bassiana* OFDH1-5 + *T. asperellum* GDFS1009, and (IC) no inoculation. All treatments except CK were infested with *O. furnacalis* larvae. Each treatment was replicated three times.

The *B. bassiana* OFDH1-5 (ACCC32726) sourced through Jilin Academy of Agricultural Sciences, Gongzhuling, China and the *T. asperellum* GDFS1009 (CGMCC NO. 9512) obtained from the School of Agriculture and Biology, Shanghai Jiao Tong University, Shanghai, China, were selected for this study based on the previous results (Batool et al., 2020). Fungal strains were grown and conidial suspensions of *B. bassiana* OFDH1-5 (1 × 10^9^), *T. asperellum* GDFS1009 (1 × 10^9^), and combined suspension of *B. bassiana* OFDH1-5 + *T. asperellum* GDFS1009 (5.9 × 10^5^ + 8.4 × 10^8^) in 1:1 were prepared as described by Batool et al. (2020). An interaction analysis to check the synergistic effect of two strains was performed based on laboratory pathogenicity analysis data (Supplementary Table 1). Surface-sterilized maize seeds were soaked in respective conidial suspension and placed in a shaker incubator (HZP-250) at 25 ± 2°C and 200 rpm for 12 h (Motholo, 2019), and then dried under a laminar air flow hood before planting (Cherry et al., 2004). For insect control-IC, control-CK, and soil drenching treatments BB-2, TH-2, and BT-2, seeds were soaked in a sterilized.1% (v/v) tween 80. For soil drenching treatments, 30 ml/plant fungal suspension was applied around the root zone after 1 week of germination.

Fifty plants per treatment were planted and replicated three times with 5 m spacing between rows. Plants of each treatment were planted 2.5 m apart from each other to hinder communication. The field conditions and management were consistent with the common local farming agricultural practices and covered with nylon netting to prevent insect immigration. All the plants selected for this experiment were healthy and developmentally similar.

### Confirmation of Endophytic Colonization of Plants

To confirm the endophytic colonization of plants by fungal stains, a re-isolation method was used. Leaves were surface-sterilized in 1% sodium hypochlorite for 3 min, 70% ethanol for 1 min followed by washing with sterilized distilled water three times. Leaves were then cut into small segments by using sterilized scissors and placed on potato dextrose agar (PDA) plates. After incubation of 3–7 days at 25 ± 2°C, the presence or absence of fungal growth from leaves was recorded and the colonization % was calculated (Quesada-Moraga et al., 2006).

### Insect Infestation and Plant Sampling

The *O. furnacalis* neonates were obtained from the Institute of Plant Protection, CAAS, and reared on an artificial diet. Insect infestation for larval survival and maize damage rating was carried out at the vegetative and silking stage, representing 1st and 2nd generation infestation, respectively. Healthy plants were infested artificially with 60 neonates (<24 h old) on June 30 and June 25 for 1st generation infestation, and on August 10 and 6 for the 2nd generation infestation in 2018 and 2019, respectively. The infestation was done during the evening to avoid exposure of neonate to direct sunlight and high temperature.

For antioxidants, chlorophyll, and molecular assays, 3rd instar larvae of *O. furnacalis* were placed on each leaf of healthy vegetative stage plants covered with small net cages to restrict their movement. Damaged leaves around the larval feeding site were collected in three replicates at 0-, 12-, 24-, 48-, and 72-h post infestation and immediately frozen in liquid nitrogen. Samples were stored at −80°C until further analysis (Guo et al., 2019).

### Maize Damage Rating and Larval Mortality

When fifth instar *O. furnacalis* larvae were observed around the maize field of 1st generation on July 28 and 23 and of 2nd generation on September 9 and 4 (ready to harvest), maize plants were dissected to record the larval mortality rate (%), plant height, tunnel number, tunnel length, kernel number kernel weight, and fungal outgrowth on larval cadavers (Xie et al., 2015).

### Measurement of Plant Antioxidants and Chlorophyll Content

Enzymatic antioxidants including POD, SOD, protease, polyphenol oxidase, proline content, and chlorophyll content were estimated in maize leaves at 0-, 12-, 24-, 48-, and 72-h post infestation. Detailed protocols are described in our previous article Batool et al. (2020).

### Transcriptome Profiling and Bioinformatics Analysis

The RNA was isolated using the TIANGEN kit (Beijing, China), following the manufacturer’s instructions. Sequencing libraries were generated using NEBNext Ultra™ RNA Library Prep Kit for Illumina (New England Biolabs, Ipswich, MA, United States) according to the manufacturer’s instructions, and index codes were added to attribute sequences of each sample. Library quality was assessed on the Agilent Bioanalyzer 2100 system. Clean reads were then mapped with the reference genome sequence by Hisat2 tools soft (Kim et al., 2015). Gene function was annotated based on database information. DEGs were identified using the DESeq2 with *P*-value < 0.05 (Wang et al., 2010). We used KOBAS software to test the statistical enrichment of DEGs in kyoto encyclopedia of genes and genomes (KEGG) pathways.

### Weighted Gene Co-expression Network Analysis for Identification of Key Candidate Genes Involved in Maize Defense

Weighted gene coexpression network analysis (WGCNA) was performed in R (version 4.1.2) using default parameters to simplify genes into expressed modules to identify the defense-related hub genes (Langfelder and Horvath, 2008). The fragments per kilobase of transcript per million mapped reads (FPKM) values were normalized, and an adjacency matrix was constructed. The plant antioxidant and the chlorophyll analysis data, used as phenotypic data, were imported into the WGCNA package, and correlation between antioxidants and chlorophyll data and gene modules were calculated using the default settings. The WGCNA package was used to convert the adjacency matrix into a topological overlap matrix (TOM). After constructing a network, the transcripts with identical expression patterns were grouped into one module, and eigengenes were also calculated for these modules. The genes from each module were exported using the default parameters for cytoscape export.

#### Validation of Intramodular Candidates Through Real Time-Quantitative Polymerase Chain Reaction

Validation of gene expression by RT-qPCR was carried out in three biological replicates on Applied Biosystems 7500 Fast Real-Time PCR System (Applied Biosystems, Foster City, CA, United States) using an SYBR Green (TAKARA Bio Inc., Japan) following the manufacturer’s guide. The RNA was isolated using a TIANGEN kit (Beijing, China) and the complementary DNAs (cDNAs) were synthesized using the One-Step gDNA Removal and cDNA Synthesis SuperMix (TransGen Biotech Co., Ltd., Beijing, China) by using the user’s manual. The amplification program followed was 95°C (15 s), followed by 40 cycles at 60°C (60 s), and 95°C (30 s). Actin (accession number-EU585777.1) was used as a reference gene. Fold changes of gene expression level were calculated using the 2^–ΔΔ*CT*^ method (Guo et al., 2019). Primers used are given in Supplementary Table 4.

### Metabolome Profiling and Quantification of Phytohormones

Metabolite extraction and analysis were performed by using ultra-high-performance liquid chromatography-mass spectroscopy (UHPLC-MS, ExionLC, AB SCIEX; Waters, Manchester, United Kingdom) by following the protocol described by Yu et al. (2020). Quantification of SA, JA, and ET was done by following Guo et al. (2021). All the samples were collected as three biological replicates.

### Data Analysis

Data collected from the maize physiological and biochemical experiments were analyzed using Statistix 8.1 software and the significance of *p* < 0.05 was applied. Alignment of RNA-seq data was done with HISAT2 software. Assembling of transcripts with mapped reads was done by using String Tie. To quantify the expression levels of transcripts for each sample, we used Asprofile software. The WGCNA was performed in R packages (version 4.1.2). Statistical comparisons of metabolite concentration were made using a SAS statistics package version 9.2 (SAS Institute Inc, 2015) and a significance level of *P* < 0.05 was applied.

## Results

### Confirmation of Endophytic Colonization of Plants

**FIGURE 1:**
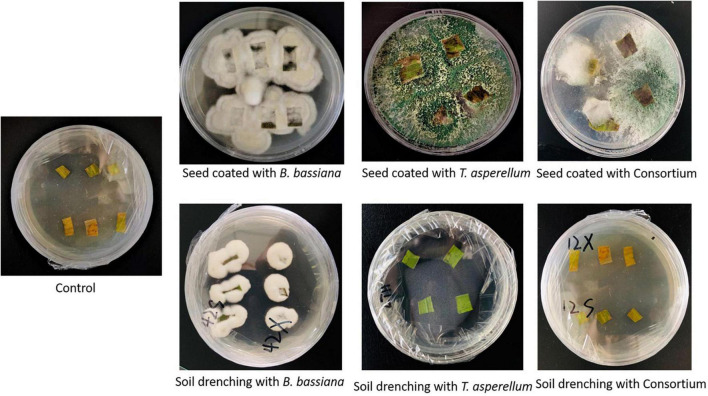
Confirmation of endophytic colonization of plants by fungal isolates through the re-isolation method.

By a re-isolation method, the mycelia growth of *B. bassiana* OFDH1-5 and *T. asperellum* GDSF1009 were observed growing from leaf segments placed in PDA plates after 3 days, whereas, in the control treatment, no fungal growth was observed. In comparison with the soil drenching treatments, 19.6 and 15.9%, 42.2 and 39.44%, and 55 and 43.17% higher growth was recorded in seed coated treatment BB-1, TH-1, and BT-1 in 2018 and 2019, respectively (Table 1 and Figure 1).

**TABLE 1:** Percentage (%) endophytic colonization of plants by fungal isolates among different treatments in 2018 and 2019.

Treatments	Endophytic colonization (%)	
	2018	2019
CK	0 ± 0^f^	0 ± 0^f^
BB-1	85.02 ± 1.67^a^	82.40 ± 0.73^b^
BB-2	68.33 ± 2.55^c^	69.28 ± 1.50^c^
TH-1	86.67 ± 0.96^a^	89.70 ± 2.01^a^
TH-2	50.01 ± 1.67^d^	54.32 ± 0.35^d^
BT-1	83.33 ± 0.96^b^	89.87 ± 1.20^a^
BT-2	46.67 ± 2.55^e^	51.07 ± 0.65^e^
IC	0 ± 0^f^	0 ± 0^f^

*Mean ± SD followed by different lowercase letters indicate significant differences among the treatments (p < 0.05). Treatments include: (CK) untreated control, (BB-1) seed treatment with B. bassiana OFDH1-5, (BB-2) soil drenching with B. bassiana OFDH1-5, (TH-1) seed treatment with T. asperellum GDFS1009, (TH-2) soil drenching with T. asperellum GDFS1009, (BT-1) seed treatment with consortium of B. bassiana OFDH1-5 and T. asperellum GDFS1009, (BT-2) soil drenching with a consortium of B. bassiana OFDH1-5 + T. asperellum GDFS1009, (IC) no inoculation.*

### Larval Survival Rate

The *B. bassiana* OFDH1-5 and *T. asperellum* GDFS1009 significantly affected the mortality rate of *O. furnacalis* larvae in both generations. In both generations, 89.38 and 92.28% of larval mortality was observed in BT-1 treatment (a consortium of both fungi) of 2018 and 2019, respectively, followed by 73.03 and 69.68% mortality was observed in BB-1, which was higher than IC. Plants inoculated with soil drenching method (BB-2, TH-2, and BT-2) showed relatively low mortality (60–65%, 40–50%, and 70–80%) in comparison with plants inoculated with seed treatment method (BB-1, TH-1, and BT-1) in 2018 and 2019, respectively (Table 2).

**TABLE 2:** Differential effect of single and consortium of *B. bassiana* OFDH1-5 and *T. asperellum* GDFS1009 inoculation on larval survival rate, number, and length of tunnels under *O. furnacalis* attack for two generations in 2018 and 2019.

Treatments	Mortality (%)		Number of tunnels/plant		Length of tunnels (cm)	
	2018	2019	2018	2019	2018	2019
**Generation 1 (vegetative stage)**						
CK	0 ± 0^h^	0 ± 0^g^	0 ± 0^f^	0 ± 0^g^	0 ± 0^f^	0 ± 0^g^
BB-1	73.03 ± 0.02^c^	69.68 ± 0.075^c^	1.033 ± 0.19^d^	1.073 ± 0.15^e^	0.652 ± 0.05^ef^	0.576 ± 0.08^e^
BB-2	61.11 ± 0.05^e^	62.43 ± 0.12^d^	1.533 ± 0.1^c^	1.66 ± 0.09^d^	1.687 ± 0.045^d^	1.319 ± 0.02^d^
TH-1	67.51 ± 0.08^d^	61.16 ± 0.14^d^	2.05 ± 0.05^b^	2.083 ± 0.1*^c^*	2.43 ± 0.12^c^	2.087 ± 0.09^c^
TH-2	52.33 ± 0.07^f^	42.57 ± 0.12^e^	2.233 ± 0.12^b^	2.56 ± 0.03^b^	4.147 ± 0.15^b^	4.125 ± 0.2^b^
BT-1	89.38 ± 0.1^a^	92.28 ± 0.03^a^	0.633 ± 0.07^e^	0.46 ± 0.06^f^	0.405 ± 0.06^f^	0.309 ± 0.03^f^
BT-2	81.47 ± 0.08^b^	71.43 ± 0.08^b^	1.383 ± 0.08^c^	1.49 ± 0.22^de^	1.185 ± 0.14^de^	1.27 ± 0.06^d^
IC	24.08 ± 0.08^g^	15.52 ± 0.46^f^	4.633 ± 0.06^a^	5.9 ± 0.3*^a^*	7.486 ± 0.6^a^	9.337 ± 0.8^a^
**Generation 2 (silking stage)**						
CK	0 ± 0^h^	0 ± 0^h^	0 ± 0^g^	0 ± 0^g^	0 ± 0^g^	0 ± 0^h^
BB-1	71.08 ± 0.06^c^	74.03 ± 0.014^c^	1.683 ± 0.12^ef^	1.676 ± 0.05^e^	3.037 ± 0.13^e^	2.547 ± 0.03^f^
BB-2	59.17 ± 0.06^e^	61.07 ± 0.05^e^	2.317 ± 0.11^d^	2.527 ± 0.02^d^	5.333 ± 0.06^d^	5.69 ± 0.1^d^
TH-1	62.45 ± 0.05^d^	65.57 ± 0.08^d^	3.3 ± 0.28^c^	3.411 ± 0.1^c^	13.993 ± 0.08^c^	11.33 ± 1.7^c^
TH-2	43.17 ± 0.1^f^	48.54 ± 0.06^f^	4.3 ± 0.22^b^	5.01 ± 0.08^b^	17.894 ± 0.8^b^	20.573 ± 0.7^b^
BT-1	86.05 ± 0.09^a^	89.67 ± 0.09^a^	1.117 ± 0.1^f^	1.25 ± 0.1^f^	1.403 ± 0.06^f^	1.103 ± 0.1^g^
BT-2	81.47 ± 0.03^b^	83.53 ± 0.05^b^	2.1 ± 0.1^de^	2.347 ± 0.09^de^	5.425 ± 0.16^d^	4.573 ± 0.67^de^
IC	16.15 ± 0.1^g^	17.07 ± 0.05^g^	6.567 ± 0.4^a^	8.267 ± 0.5*^a^*	20.055 ± 0.83^a^	25.097 ± 0.9^a^

*Mean ± SD followed by different lowercase letters indicate significant differences among the treatments (p < 0.05). Treatments include: (CK) untreated control, (BB-1) seed treatment with B. bassiana OFDH1-5, (BB-2) soil drenching with B. bassiana OFDH1-5, (TH-1) seed treatment with T. asperellum GDFS1009, (TH-2) soil drenching with T. asperellum GDFS1009, (BT-1) seed treatment with consortium of B. bassiana OFDH1-5 and T. asperellum GDFS1009, (BT-2) soil drenching with a consortium of B. bassiana OFDH1-5 + T. asperellum GDFS1009, (IC) no inoculation.*

### Maize Growth and Damage Rating

The consortium of *B. bassiana* OFDH1-5 + *T. asperellum* GDFS1009 has a positive effect on plant growth and damage rating. In Generation 1, the highest number of tunnels and tunnel lengths were recorded in the insect control treatment (IC), whereas this damage was reduced up to 86.3 and 94.6% in BT-1 in 2018, and 92.2 and 96.7% in 2019, respectively. The second lowest damage was recorded in BB-1 with 77.7 and 81.8% reduction in the number of tunnels and 91.3 and 93.8% reduction in tunnel lengths in 2018 and 2019, respectively. Similar results were recorded in generation 2 with the highest number of tunnels and tunnel lengths in IC and lowest (82.9 and 93% in 2018, and 84.9 and 95.6% in 2019) in BT-1 (Table 2).

Over the 2-year average, plant heights were reduced up to 25.65% in the insect control treatment (IC) compared to the untreated control, while application of *B. bassiana* OFDH1-5 and *T. asperellum* GDFS1009 reduced the negative effect of *O. furnacalis* feeding and increased the plant heights up to 30.65, 29.1, and 34.5% in BB-1, TH-1, and BT-1, respectively. Less effect on plant height was seen in plants inoculated with the soil drenching method (Table 3). Additionally, the application of entomopathogenic fungi significantly increased the grain yield. The maximum increase in the average number of kernels per ear was recorded as 55.2 and 70.9% in BT-1, followed by 53.4 and 69.1% in BB-1, and 47.9 and 56.6% in TH-1 in 2018 and 2019, respectively. Similar patterns of increase in weight per 100 kernels were observed with 53.3 and 79.8% in BT-1, 46.2 and 65.7% in BB-1, and 36.1 and 43.4% in TH-1 in 2018 and 2019, respectively. In contrast, the maximum reduction in grain yield was recorded in insect control (IC) treatment with 20.6 and 29.1% in the number of kernels per ear, and 21.1 and 32.1% in weight per 100 kernels in the year 2018 and 2019, respectively, compared to untreated control (CK) (Table 3).

**TABLE 3:** Differential effect of single and consortium of *B. bassiana* OFDH1-5 and *T. asperellum* GDFS1009 inoculation on maize yield under *O. furnacalis* attack in 2018 and 2019.

Treatments	Kernel number/ear		100 kernel weight		Height (cm)	

	2018	2019	2018	2019	2018	2019
CK	692.78 ± 0.5^g^	706.03 ± 0.7^g^	20.56 ± 0.5^c^	21.27 ± 0.8^c^	316.62 ± 0.5^a^	311.34 ± 1.38^b^
BB-1	843.3 ± 0.8^a^	846.27 ± 0.29^b^	23.92 ± 0.6^a^	23.95 ± 0.56^b^	298.43 ± 1.13^e^	304.27 ± 0.27^c^
BB-2	793.63 ± 0.6^e^	789.15 ± 0.5^e^	21.03 ± 0.19^bc^	21.65 ± 0.50^c^	279.61 ± 1.2^f^	296.05 ± 1.23^e^
TH-1	813.68 ± 1.25^c^	793.93 ± 1.0^d^	22.27 ± 0.45^b^	20.72 ± 0.65^cd^	307.07 ± 0.4^c^	298.43 ± 1.13^e^
TH-2	780.23 ± 0.4^f^	740.81 ± 0.7^f^	20.44 ± 0.5^c^	17.83 ± 0.5^e^	301.46 ± 1.2^d^	279.50 ± 0.6^f^
BT-1	853.23 ± 0.9^b^	855.3 ± 0.7^a^	25.06 ± 0.11^a^	25.98 ± 0.52^a^	311.34 ± 1.4^b^	316.62 ± 0.5^a^
BT-2	797.92 ± 0.61^d^	801.63 ± 0.5^c^	21.82 ± 0.7^bc^	23.21 ± 0.2^b^	300.23 ± 0.8^d^	300.25 ± 0.8^d^
IC	549.8 ± 1.03^h^	500.41 ± 1.01^h^	16.36 ± 0.3^d^	14.45 ± 0.50^f^	238.74 ± 1.2^g^	228.38 ± 1.01^g^

*Mean ± SD followed by different lowercase letters indicate significant differences among the treatments (p < 0.05). Treatments include (CK) untreated control, (BB-1) seed treatment with B. bassiana OFDH1-5, (BB-2) soil drenching with B. bassiana OFDH1-5, (TH-1) seed treatment with T. asperellum GDFS1009, (TH-2) soil drenching with T. asperellum GDFS1009, (BT-1) seed treatment with consortium of B. bassiana OFDH1-5 and T. asperellum GDFS1009, (BT-2) soil drenching with a consortium of B. bassiana OFDH1-5 + T. asperellum GDFS1009, (IC) no inoculation.*

**FIGURE 2:**
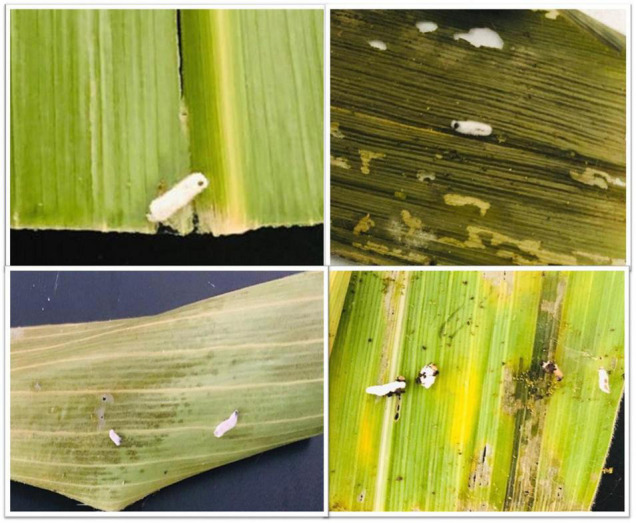
Field collected dead larvae showing the existence of fungal outgrowth from the larval cadavers.

Larval cadavers were collected from the field to determine the percentage (%) of cadavers showing a fungal outgrowth collected from the field. The highest number of cadavers (%) with fungal outgrowth was observed as 87.33 and 91.83% in seed treated consortium treatment (BT-1) in 2018 and 2019, respectively, at Generation 1 infestation. Similarly, in Generation 2 infestation, 89.68 and 84.07% cadavers were detected in BT-1 with fungal outgrowth in 2018 and 2019, respectively, followed by 71–74% in seed treated with *B. bassiana* treatment (BB-1) (Table 4 and Figure 2). Based on agronomic field data, samples from BB-1, TH-1, BT-1 (inoculated through seed treatment method), and IC were used for further analysis.

**TABLE 4:** Percentage (%) of cadavers showing fungal outgrowth collected from the field.

Treatments	Cadavers with fungal outgrowth (%)	
	2018	2019
**Generation 1 (vegetative stage)**		
CK	0 ± 0^f^	0 ± 0^e^
BB-1	74.00 ± 0.67^b^	71.68 ± 0.75^b^
BB-2	54.11 ± 2.05^d^	62.93 ± 0.23^c^
TH-1	54.67 ± 0.78^d^	59.36 ± 0.34^c^
TH-2	20.08 ± 0.477^e^	32.37 ± 1.12^d^
BT-1	87.33 ± 1.13^a^	91.83 ± 2.01^a^
BT-2	58.67 ± 1.08^c^	61.39 ± 1.08^c^
IC	0 ± 0^f^	0 ± 0^e^
**Generation 2 (silking stage)**		
CK	0 ± 0^g^	0 ± 0^g^
BB-1	71.58 ± 0.35^b^	74.32 ± 0.24^b^
BB-2	56.67 ± 0.76^cd^	61.75 ± 1.15^c^
TH-1	49.45 ± 1.35^e^	47.57 ± 0.48^e^
TH-2	24.17 ± 1.71^f^	28.50 ± 1.06^f^
BT-1	89.65 ± 1.09^a^	84.07 ± 0.19^a^
BT-2	59.97 ± 0.53^c^	57.09 ± 0.35^d^
IC	0 ± 0^g^	0 ± 0^g^

*Mean ± SD followed by different lowercase letters indicate significant differences among the treatments (p < 0.05). Treatments include (CK) untreated control, (BB-1) seed treatment with B. bassiana OFDH1-5, (BB-2) soil drenching with B. bassiana OFDH1-5, (TH-1) seed treatment with T. asperellum GDFS1009, (TH-2) soil drenching with T. asperellum GDFS1009, (BT-1) seed treatment with consortium of B. bassiana OFDH1-5 and T. asperellum GDFS1009, (BT-2) soil drenching with a consortium of B. bassiana OFDH1-5 + T. asperellum GDFS1009, (IC) no inoculation.*

### Transcriptome Responses Triggered by *B. bassiana* OFDH1-5 and *T. asperellum* GDFS1009

**FIGURE 3:**
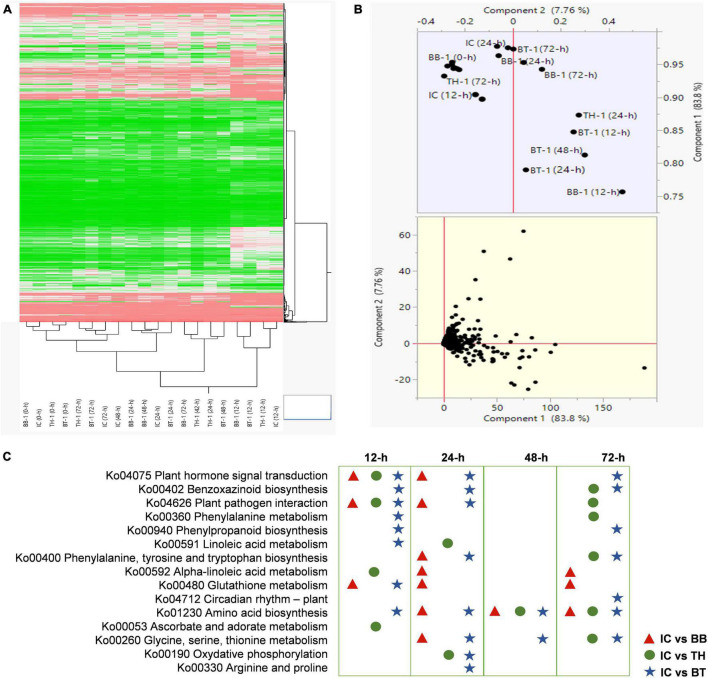
Transcriptome response of maize inoculated with single and consortium of *Beauveria bassiana* OFDH1-5 and *Trichoderma asperellum* GDFS1009 to *Ostrinia furnacalis*. **(A)** Hierarchical Cluster dendrogram of differentially expressed genes, the difference in color indicate high (red) and low (green) expression. **(B)** PCA plot showing variability of transcriptome data; **(C)** top enriched resistance related pathways in IC vs. BB, IC vs. TH, IC vs. BT at 12-, 24-, 48-, and 72-h.

For transcriptome profiling, four treatments (BB-1, TH-1, BT-1, and IC) in three replicates at 0-, 12-, 24-, 48-, and 72-h post *O. furnacalis* infestation were used to construct 60 cDNA libraries. A total of 458.04 Gb clean data was obtained and the clean data of each sample reached 5.71 Gb, and the Q30 base percentage was 93.24% and above. Clean reads were aligned with a designated reference genome (ZmB73_Ref-Gen_v4) and the comparison efficiency ranged from 79.13 to 89.36% (Supplementary Table 2). Based on the comparison results, the gene expression level analysis was performed and the differentially expressed genes were identified according to their expression levels | log2 (fold-change)| > 1 and an adjusted *P*-value < 0.05 in each pairwise comparison. The abundance of upregulated genes was higher than downregulated genes compared to all-time points (Supplementary Table 3). Identified genes in all samples were hierarchically clustered relative to control. The difference in color indicates high (red) and low (green) expressions (Figure 3A). The principal component analysis revealed the variability among transcriptome data of different samples. Clustering of samples away from each other and control group (IC) indicates a fungal inoculation and *O. furnacalis*-induced changes in gene expression specially in consortium treatment (BT) (Figure 3B). In total, 13,156 common DEG’s were identified in all the combinations at different time points.

To explore the response of different processes in combined *B. bassiana* OFDH1-5 and *T. asperellum* GDFS1009 treatments, we analyzed the KEGG enrichment analysis of common DEG’s. The sets of DEG’s were assigned to significant KEGG Pathways (*P* > 0.05) (Supplementary Figure 1), and the top enriched resistance related pathways in IC vs. BB, IC vs. TH, and IC vs. BT at 12-, 24-, 48-, and 72-h were listed in Figure 3C, which show uniquely induced genes enriched in a higher number of metabolic processes in consortium treatment (BT).

### *B. bassiana* OFDH1-5 and *T. asperellum* GDFS1009 Triggered Antioxidants and Chlorophyll Content in *O. furnacalis-Induced Defense*

**FIGURE 4:**
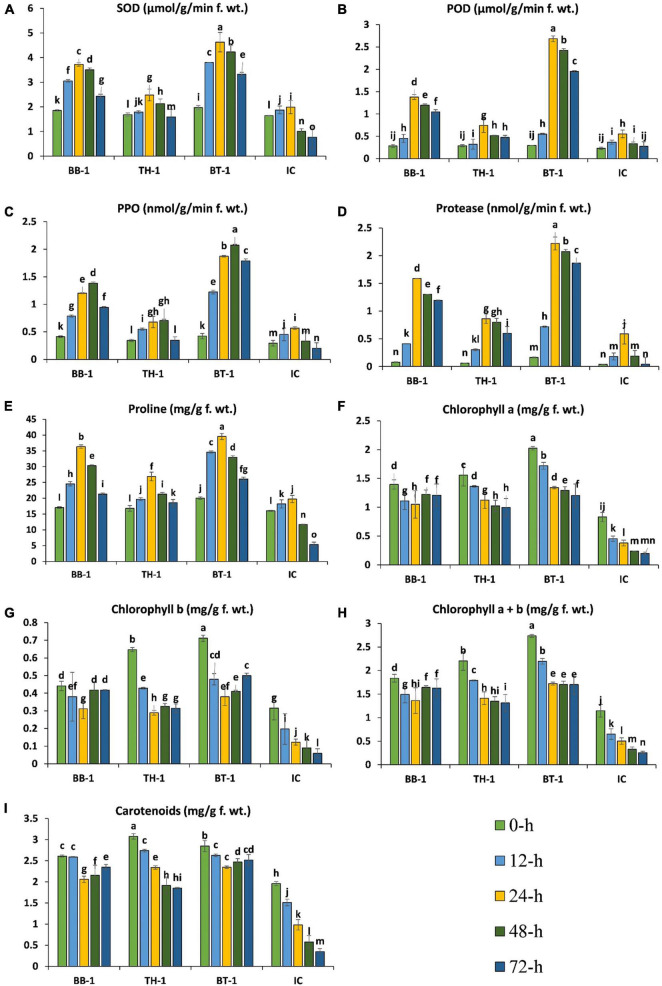
*Beauveria bassiana* OFDH1-5 and *T. asperellum* GDFS1009 induced response of maize antioxidants and chlorophyll content under *O. furnacalis* attack. **(A)** Superoxide dismutase (SOD); **(B)** peroxidase (POD); **(C)** polyphenol oxidase (PPO); **(D)** protease; **(E)** proline; **(F)** chlorophyll a; **(G)** chlorophyll b; **(H)** chlorophyll a + b; **(I)** carotenoids. Different lowercase letters above each bar indicate significant differences among treatments (*p* < 0.05). Treatments include (BB-1) seed treatment with *B. bassiana* OFDH1-5, (TH-1) seed treatment with *T. asperellum* GDFS1009, (BT-1) seed treatment with a consortium of *B. bassiana* OFDH1-5 and *T. asperellum* GDFS1009, (IC) no inoculation.

The antioxidant enzyme activities started to increase at 12-h in all the infested treatments and reached a maximum point at 24-h of insect feeding and gradually decreased until 48-h, while polyphenol oxidase (PPO) reached the maximum at 48-h. The production of antioxidant enzymes was further triggered by entomopathogenic fungal treatments and the highest productions were observed in consortium treatment (BT-1). An increase of 132. 5-, 385. 3-, 100. 6-, and 277.7-folds were recorded in SOD, POD, proline, and protease activities at 24-h of *O. furnacalis* feeding. In the case of PPO, a 522.7-fold increase was recorded at 48-h and the lowest enzyme activities were observed in IC treatment (Figures 4A–E).

Chlorophyll content was observed to be gradually decreased from 0-h to 72-h due to *O. furnacalis* feeding, but fungal inoculation increased the chlorophyll content. The maximum content of chlorophyll-a and chlorophyll-b were recorded in BT-1, followed by TH-1, and BB-1 compared to insect control (IC). Chlorophyll-a, chlorophyll-b, and carotenoid were reduced up to 80-, 86-, and 86.3-folds, respectively, in the insect control (IC) (Figures 4F–I).

### Co-expression Network Analysis for the Identification of Hub Genes Involved in Maize Defense

**FIGURE 5:**
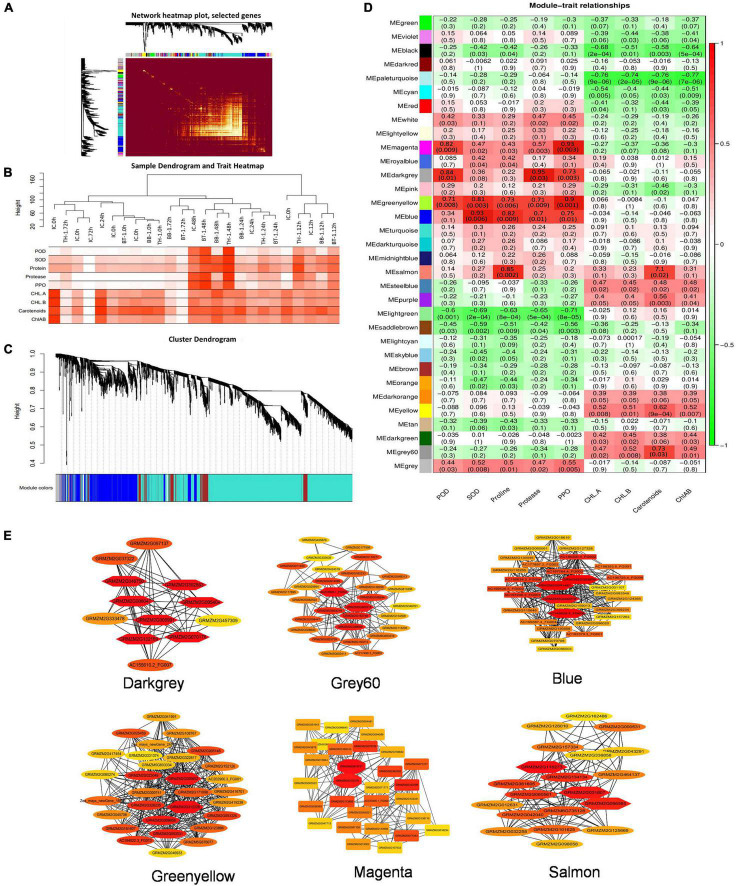
Weighted gene coexpression network analysis (WGCNA) analysis to identify key candidate genes involved in maize defense. **(A)** Cluster dendrogram and network heatmap of genes subjected to coexpression module calculation. **(B)** Sample dendrogram and module trait heatmap of each time point. **(C)** Hierarchical clustering presenting eleven modules having coexpressed genes. Each leaflet in the tree corresponds to an individual gene. **(D)** Module-trait associations based on Pearson correlations. The color key from green to red represents *r*^2^ values ranging from –1 to 1. **(E)** Gene networks of six highly correlated modules with phenotypic traits. Genes in different red shape due to the highest weight within the module represents hub genes (key candidate genes) and are coded for gene descriptors based on annotation. Treatments include (BB-1) seed treatment with *B. bassiana* OFDH1-5, (TH-1) seed treatment with *T. asperellum* GDFS1009, (BT-1) seed treatment with a consortium of *B. bassiana* OFDH1-5 and *T. asperellum* GDFS1009, (IC) no inoculation.

The FPKM values of 13,156 common DEGs and plant biochemical parameters (SOD, POD, proline, protease, PPO, Chl-a, chl-b, carotenoids, and chl-a + b) at different time points were used for the weighted gene coexpression network analysis and the module trait correlations were calculated. Thirty-three gene modules were identified based on coexpression patterns. Each module is represented by a different color and presented as a cluster dendrogram and network heatmap (Figures 5A–D).

Among the 33 identified modules, only six showed significant correlations with the phenotypic data. The blue module with 326 genes showed significant correlations with SOD, proline, protease, and PPO with correlation coefficients (*r*^2^) of 0.93, 0.82, 0.7, and 0.75, respectively. The darkgrey module possessing 149 genes showed positive correlations with POD (*r*^2^ = 0.84), PPO (*r*^2^ = 0.73), and protease (*r*^2^ = 0.95). The green-yellow module, having 223 genes, showed significant positive correlations with POD (*r*^2^ = 0.71), SOD (*r*^2^ = 0.81), proline (*r*^2^ = 0.73), protease (*r*^2^ = 0.71), and PPO (*r*^2^ = 0.9). Grey60 module consists of 57 genes, and it showed a positive correlation with carotenoids (*r*^2^ = 0.73). Magenta module with 98 genes showed positive correlations with POD (*r*^2^ = 0.82) and with PPO (*r*^2^ = 0.93). Salmon module with 145 genes showed a correlation with proline (*r*^2^ = 0.85) (Figure 5D). The hub genes from these modules were selected by the cytoscape built-in extension, namely “CytoHubba,” visualizing the gene networks (Figure 5E). Furthermore, to find out the genes involved in maize defense within these gene networks, gene annotation information was extracted from maize reference genome (ZmB73_Ref-Gen_v4).

#### Real Time-Quantitative Polymerase Chain Reaction Validation of Intramodular Hub Genes

**FIGURE 6:**
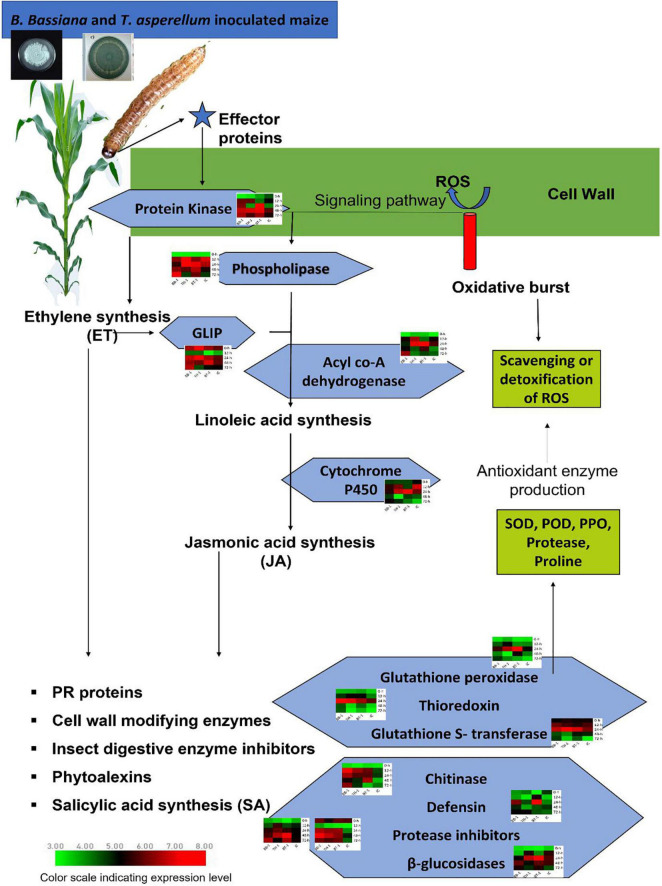
Schematic representation of transcriptional strategies employed by maize inoculated with a consortium of *B. bassiana* OFDH1-5 and *T. asperellum* GDFS1009 compared with single inoculation to induce enhanced expression of key genes to involve in initiating signaling pathway to activate phytohormone biosynthesis pathways which regulate the production and expression of defense genes to induce maize resistance against *O. furnacalis*. Treatments include (BB-1) seed treatment with *B. bassiana* OFDH1-5, (TH-1) seed treatment with *T. asperellum* GDFS1009, (BT-1) seed treatment with a consortium of *B. bassiana* OFDH1-5 and *T. asperellum* GDFS1009, (IC) no inoculation.

Selected hub genes were further validated by performing RT-qPCR at different time points, thus, narrowing down the number of selected genes. Finally, from 28 hub genes, we selected 13 key candidate genes with stable and consistent expressions responsible for maize defense against *O. furnacalis* (Figure 6). The RT-qPCR results were consistent with the RNA-seq data (Supplementary Figure 2). All the key candidate genes, along with other hub genes, are shown in Table 5.

**TABLE 5:** Selected hub genes from all coexpressed modules with functional annotation.

Gene ID	Module	Annotation	Identified as key candidate gene
GRMZM2G144153	Darkgrey	Glutathione peroxidase	✓
AC212068.4_FG005	Darkgrey	GDSL-like lipase	✓
GRMZM2G046750	Darkgrey	Protease inhibitor	✕
GRMZM2G392863	Darkgrey	Defensin like protein	✓
GRMZM2G004466	Darkgrey	Protease inhibitor	✓
GRMZM2G005991	Darkgrey	Protease inhibitor	✕
GRMZM2G132169	Darkgrey	Laccase (L-ascorbate oxidase precursor)	✕
GRMZM5G865319	Grey60	Serpin	✕
AC233926.1_FG002	Grey60	Protease inhibitor	✓
GRMZM2G171444	Yellow	Ribosomal protein L19	✕
AC200099.4_FG006	Blue	Cytochrome P450	✕
GRMZM2G025459	Blue	Protein kinase	✓
GRMZM2G091481	Blue	Thioredoxin	✓
GRMZM5G864319	Blue	Acyl-CoA dehydrogenase	✓
GRMZM2G035708	Darkgreen	Photosynthetic NDH subunit	✕
GRMZM2G453805	Green yellow	Chitinase	✓
GRMZM2G154523	Green yellow	Patatin-like phospholipase	✓
GRMZM2G031660	Magenta	Beta-glucosidase 18	✓
GRMZM2G139874	Magenta	Cytochrome P450	✓
GRMZM2G023152	Purple	*O*-methyltransferase ZRP4	✕
AC225718.2_FG009	Purple	Wound-induced protein	✕
GRMZM2G116273	Salmon	Glutathione *S*-transferase	✓
GRMZM2G031607	Salmon	SGS domain-containing protein	✕
GRMZM2G096585	Salmon	peptidyl-prolyl isomerase	✕
GRMZM2G310368	Steel blue	Ethylene-responsive transcription factor	✕
Zea_mays_newGene_38529	Steel blue	Plant transposase	✕
Zea_mays_newGene_15021	Steel blue	Plant transposase	✕
Zea_mays_newGene_17096	White	Ribosomal protein S19e	✕

### *B. bassiana* OFDH1-5 and *T. asperellum* GDFS1009-Enhanced Expression of *Key Candidate Genes in Maize Defense Response* Against *O. furnacalis* Herbivory

Three key genes in the blue module were identified as protein kinase gene (GRMZM2G025459), which participates in the phytohormone signaling pathway to initiate a defense response and to regulate the expression of genes involved in ion homeostasis and oxidative stress responses, acyl-CoA dehydrogenase (GRMZM5G864319), which is involved in an alpha-linolenic acid metabolism of jasmonic acid pathway and thioredoxin gene (GRMZM2G091481), which acted as an important component of the signaling pathway in plant antioxidant networks. Similarly, in the salmon module, the Glutathione *S*-transferase was identified as a key gene (GRMZM2G116273), which plays a crucial role in plant oxidative stress response against high ROS (reactive oxygen species) levels generated by the insect attack.

Four genes, (GRMZM2G144153, AC212068.4_FG005, GRMZM2G392863, and GRMZM2G004466) were identified as key genes in darkgrey module. These genes were identified as glutathione peroxidase, GDSL- like lipase, defensin, and protease inhibitor. Glutathione peroxidase gene (GRMZM2G144153) is known to be activated upon a cell wall damage by insect feeding. In addition, it is also involved in the ROS scavenging, lignification of the cell wall, and synthesis of phenolic compounds. The GDSL- like lipase (AC212068.4_FG005) acts as an elicitor of systemic resistance linked with ET pathways. Upon insect feeding, its expression depending upon the ethylene signaling generates the systemic signals which translocate to other healthy parts of plants and propagate a systemic resistance throughout the plant. The defensin gene (GRMZM2G392863) has a great insecticidal property of inhibiting insect digestive enzymes; α-amylase play a crucial role in breaking down plant starch and hydrolyze proteins. Protease inhibitor genes (GRMZM2G004466) were characterized as key genes. One more protease inhibitor gene (AC233926.1_FG002) was identified in the grey60 module as a key gene. They were found to participate in the reduction of insect growth, and, thereby, suppress the infection process by inhibiting extracellular proteases released by insects.

Patatin-like phospholipase gene (GRMZM2G154523) is responsible for the synthesis of linolenic acid, as a precursor of jasmonic acid biosynthesis pathway and chitinase gene (GRMZM2G453805), expressed by plants and acted as a self-defense mechanism against insect attack, were found as defense-related hub genes in the green-yellow module.

Beta-glucosidase 18 and cytochrome p450 (GRMZM2G031660 and GRMZM2G139874), which participate in various detoxification and biosynthetic pathways to protect the plant, were found as key genes in the magenta module. All genes showed differential expression patterns among all fungal-inoculated treatments, with the highest expression levels at 24- and 48-h post larval infestation specially in BT-1. The role and expression patterns of all key genes in maize defense response are shown in Table 5, Figure 6, and Supplementary Figure 2.

### Metabolomic Adjustments Triggered by *B. bassiana* OFDH1-5 and *T. asperellum* GDFS1009

**FIGURE 7:**
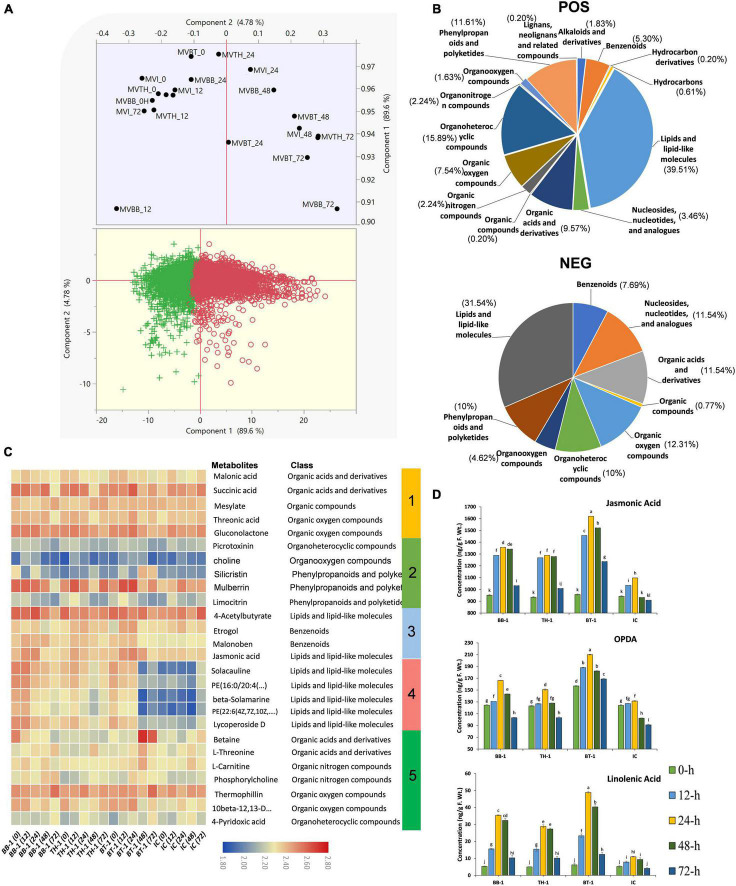
Metabolome profiling of maize inoculated with single and consortium of *B. bassiana* OFDH1-5 and *T. asperellum* GDFS1009 and subjected to *O. furnacalis* attack. **(A)** PCA plot show divergence in metabolome data among treatments (MVBB, MVTH, MVBT, and MVI stands for BB-1, TH-1, BT-1, and IC, respectively). **(B)** Pie chart showing the percentage distribution of differentially annotated metabolites in different classes. **(C)** Hierarchical clustering of identified abundant metabolites. **(D)** Concentration of phytohormones in maize plants. Data is mean of three replicates and different lower-case letters above each bar indicate significant differences among treatments (*p* < 0.05). Treatments include (BB-1) seed treatment with *B. bassiana* OFDH1-5, (TH-1) seed treatment with *T. asperellum* GDFS1009, (BT-1) seed treatment with a consortium of *B. bassiana* OFDH1-5 and *T. asperellum* GDFS1009, (IC) no inoculation.

To obtain complete insights of *B. bassiana* OFDH1-5 and *T. asperellum* GDFS1009 inoculated maize under the *O. furnacalis* attack, we performed a comprehensive untargeted metabolomic analysis of all BB-1, TH-1, BT-1, and IC at 0-, 12-, 24-, 48-, and 72-h by UHPLC-QTOF-MS for the first time. The principal component analysis (PCA) analysis clearly showed the diversity among samples. Interestingly 0-h samples grouped indicating less diversity and other samples clustering away from control showed diversity in metabolomic data (Figure 7A). Through electrospray ionization (ESI) total of 7,367 NEG (negative) and 13,183 POS (positive) mass/retention time features were detected (Supplementary Data 4), and further 130 NEG and 491 POS differentially annotated and abundant metabolomic features were filtered based on different classes (Supplementary Data 5 and Figure 7B). Within the POS metabolic features, 9 fall in alkaloids and derivatives class, 26 in benzenoids, 3 in hydrocarbons, 194 in lipids and lipid-like molecule, 17 in nucleosides, nucleotides, and analogs, 47 in organic acids and derivatives, 11 in organic nitrogen compounds, 37 in organic oxygen compounds, 78 in organoheterocyclic compounds, 8 in organooxygen compounds, 57 in phenylpropanoids and polyketides and 1 each in hydrocarbon derivatives, organic compounds, organonitrogen compounds and lignans, neolignans, and related compounds. In the case of NEG metabolic features, 10 fall in benzenoids class, 15 each in nucleosides, nucleotide and analogs class and organic acid and derivative class, 1 in organic compounds, 16 in organic oxygen compounds, 13 in organoheterocyclic compounds, 6 in organooxygen compounds, 13 in phenylpropanoids and polyketides, and 41 in lipids and lipid-like molecules class (Figure 7B). Through hierarchical clustering, metabolites were divided into five clusters. Obvious changes were seen in the abundance of all metabolites belonging to different classes with a higher abundance of malonic acid, succinic acid, threonic acid, and gluconolactone in cluster 1, silicristin and mulberrin in cluster 2, etrogol and jasmonic acid in cluster 3, and betaine, L-threonine, L-carnitine, and thermophilin in cluster 5 were observed in a consortium treatment (BT-1). In cluster 4, all inoculated treatments have shown higher abundance as compared to control (IC) (Figure 7C).

### *B.bassiana* OFDH1-5 and *T. asperellum* GDFS1009-Enhanced Phytohormone Concentration Induced by *O. furnacalis* Herbivory

To determine changes in stress-related phytohormones, we quantified JA, ET, and SA. A significant increase in the contents of phytohormones was recorded in all fungal inoculated treatments, but highest in consortium treatment (BT-1) at 24-h post infestation with an increase of 32.2, 37.4, and 77.5% in JA, SA, and ET, respectively (Figure 7D).

## Discussion

*Ostrinia furnacalis* is one of the most destructive insect pests of maize and it is responsible for major yield losses. It causes major damage at whorl stage plants by feeding in leaves and boring in maize stalk. Besides other pest control strategies, the biological control approach is of the highest interest because of its eco-friendly nature. The latest research proved that a consortium of biological control agents was effective against maize pests (Sarma et al., 2015; Batool et al., 2020). In our previous study (Batool et al., 2020), we have investigated and concluded that the entomopathogenic strain, *B. bassiana*, and plant pathogen antagonistic strain, *T. asperellum*, have both strong synergistic potential and can be used in the consortium to enhance their biological control activity. They inhibit the insect survival and damage on maize plants by inhibiting the immune response of *O. furnacalis* at the transcriptome level. Based on our previous findings, in the present investigation, we further investigated the effect of *B. bassiana*, *T. asperellum*, and their consortium on insect survival, maize damage, and growth, at field level under natural environmental conditions. Furthermore, WGCNA was used for the first time in an entomopathogenically inoculated maize to integrate transcriptome and biochemical data and an untargeted metabolite profiling at the vegetative stage of maize to identify the candidate genes and metabolites associated with the regulation of defensive biochemical enzymes, and induction of maize resistance and maize defense pathways against *O. furnacalis*.

Both entomopathogenic/antagonistic fungi can colonize the plant endophytically and can induce a defensive response upon insect feeding as a challenge, thereby killing or arresting the growth of insects (Batool et al., 2020). Plants can provide a suitable environment for entomopathogenic fungi and their insecticidal activity can suppress the infection and facilitate plants by increasing insect resistance and plant growth (Gupta et al., 2016). Plant colonization largely depends upon the method of application and the seed treatment method was proven to be more effective (Batool et al., 2020). The seed treatment has the advantage of colonizing radical and plumule at the same time, as they are close to seed and can colonize the whole plant during its growth (Muvea et al., 2014). Our results were also in accordance with the findings of pathogenicity against *O. furnacalis* and reducing its survival, tunnel number and length, and increasing grain yield and plant growth in the consortium-treated Plants (BT-1). Some previous research also showed that the growth of entomopathogenic fungi in plants can prevent plant herbivory, and the *B. bassiana* have insecticidal activity against several insects including corn borers (Wagner and Lewis, 2000). *Trichoderma* is generally known for the function to induce the ISR of the whole maize plant against plant diseases and pest insects through colonization of maize roots (Rivas-Franco et al., 2020). The activity of *T. asperellum* was relatively low as compared to others, but its pathogenic activity was enhanced by the co-cultivation with *B. bassiana*. Shakeri and Foster (2007) also stated that the *Trichoderma* pathogenicity against borer larvae was lower and it can be increased by modifying them at the genetic level. Trichoderma could generate synergistic action with other biocontrol organisms, including the entomopathogenic fungi (Netzker et al., 2015), describing the use of consortium technique to enhance the activity through intercommunication.

In response to an insect attack, plants produce several defensive antioxidants including, SOD, POD, protease, PPO, and proline to scavenge ROS. These enzymes were considered the most important plant defense-related enzymes against herbivores (Guo et al., 2017; Batool et al., 2020). We also observed that antioxidant activities such as SOD, POD, PPO, protease, and proline were increased in all insect-infested plants, with a maximum increase at 24-h post infestation, while these enzyme activities were relatively higher in entomopathogenic fungal inoculated plants. The results revealed that these biocontrol agents enhanced the defense mechanism in plants by enhancing the production of defensive enzymes. Consortium treatment showed a maximum increase among all treatments. Some previous studies also reported the induction of SOD, POD, proline, protease, and PPO by biological control agents (Bano and Muqarab, 2017). PPO and protease play a defensive role against insect attack and induce resistance in plants (Joe and Muthukumaran, 2008). The chlorophyll content is one of the important components for plant growth and we observed that the chlorophyll content was increased in plants treated with entomopathogenic fungi compared to the insect control (IC). The same trend was recorded in previous studies (Bano and Muqarab, 2017; Batool et al., 2020). SOD is considered as the first enzyme in the scavenging process of ROS and its augmented scavenging action and detoxified by POD and act as a signaling agent to activate defensive genes in plants (Bano and Muqarab, 2017). Proline is best known as hydroxyl radical scavenger also serves as an energy source in plants. PPO is an important enzyme, which induces insect resistance in plants by participating in cell lignification and oxidation of polyphenols. Protease also has a defensive role against insect attacks (Batool et al., 2020).

With rapid development in bioinformatics, the weighted gene co-expression analysis (WGCNA) approach has been developed to explore the functionality of transcriptome. WGCNA was used to detect the genes which co-express (Lin et al., 2019). The relationship between trait and module identifies the most vital hub genes, which supports an efficacious way to explore the in-depth mechanism of complex traits (Lv et al., 2020). Considering the importance of fungal inoculants against the control of *O. furnacalis*, plant yield, and maize defense through augmented production of enzymatic and non-enzymatic antioxidants. We also integrated these biochemical enzymes and compounds with plant transcriptome at 0-, 12-, 24-, 48-, and 72-h. We performed a transcriptome-based analysis to identify DEGs in different combinations. The DEGs were then analyzed by WGCNA along with biochemical parameters and 33 gene modules represented by different colors were identified. Among them, 6 were significantly correlated with SOD, POD, PPO, protease, proline, and chlorophyll content. All genes contained in modules were functionally annotated to identify hub genes and 28 hub genes were identified and validated through RT-qPCR. Finally, 13 genes were characterized as the key candidates that are involved in maize defense mechanism against *O. furnacalis* based on significant correlation of hub genes from the module with biochemical enzymes and chlorophyll content, and expression pattern of selected genes. Gamboa-Tuz et al. (2018) also identified stress related hub genes from papaya leaves and roots. Pan et al. (2020) correlated transcriptome through WGCNA to identify key genes in maize against herbivore.

### Activity of Identified Candidate Genes in Phytohormone Signaling and Biosynthesis Pathways

Insect herbivores induce the expressions of genes associated with the phytohormone signaling pathway (Li et al., 2016). Plant recognizes herbivore damage as a primary signal and triggers the expression of defense related genes. Initially, when an insect starts feeding, the plant reacts to it by producing ROS and effector proteins to activate the expression of signaling molecules and initiate signaling pathways of systemic resistance (Almagro et al., 2009). We identified four such signaling related genes; protein kinase (GRMZM2G025459), GDSL-like Lipase (AC212068.4_FG005), Patatin-like phospholipase (GRMZM2G154523), and Cytochrome P450 (GRMZM2G139874), which showed a high response against *O. furnacalis* feeding in fungal inoculated treatments. The activity of GDSL- like lipase (GLIP) is highly dependent on the Ethylene ET- the pathway to generate systemic signals to produce GLIP in the uninfected part of the plants to protect it from the insect (Kwon et al., 2009). They also reported the reduction in the growth of *Pseudomonas syringae* by the accumulation of GLIP 1 in all parts of the plant. Patatin like phospholipase is a signaling molecule responsible for the release of linolenic acid from phospholipids to biosynthesize jasmonic acid (JA) (Ryu, 2004). Meijer and Munnik (2003) observed the presence of phospholipases in wounded tomatoes and many other plants. Dhondt et al. (2002) also found three lipase genes in response to external elicitor or infection before the accumulation of JA. Acyle co-A dehydrogenase gene (GRMZM5G864319) was also identified as a candidate gene that participates in the linolenic acid metabolism of the JA biosynthesis pathway (Tang et al., 2015). Cytochrome P450 (CYP) participates in plant growth promotion and protection from biotic and abiotic stresses through detoxification and biosynthesis pathways. To date, the involvement of cytochrome p450 in different metabolic pathways including allene oxide synthesis in the jasmonic acid pathway, hormone metabolism, and biosynthesis of phytoalexin has been confirmed (Jun et al., 2015). *CYP74A* (allene oxide synthase, AOS) catalyzes the dehydration of hydroperoxide to an instable allene oxide in the JA biosynthetic pathway and plays an important role in wound-induced defense against biotic attacks (Park et al., 2002).

### Gene Expression Regulating Antioxidant Enzyme Production

Biosynthesis of JA- and ET- activate the production of PR-proteins, cell wall modifying enzymes, salicylic acid synthesis, and phytoalexins as a defense response to induce systemic resistance. In the present investigation, the genes encoding defensive enzymes and proteins such as Thioredoxin (GRMZM2G091481), Glutathione Peroxidase (GRMZM2G144153), Glutathione *S*-transferase (GRMZM2G116273), Defensin like protein (GRMZM2G392863), protease inhibitor (GRMZM2G004466), protease inhibitor (AC233926.1_FG002), Chitinase (GRMZM2G453805), Beta-glucosidase 18 (GRMZM2G031660) were upregulated with higher expressions in consortium treatment (BT-1). The expression patterns of these genes coincide with the levels of biochemical enzymes produced in all treatments, which indicates that the over expressions of these genes may regulate higher production of enzymatic (SOD, POD, PPO, and Protease) and non-enzymatic (proline) antioxidants to initiate maize defense responses, such as lignification, suberization, insect growth inhibition, and scavenging of ROS.

#### Genes Mediating Redox Signaling

Enzymatic and non-enzymatic antioxidants reduce the level of ROS and SOD is considered as the first and most important enzyme in the scavenging process as it catalyzes O_2_^–^ into H_2_O_2_. The accumulation of the highest H_2_O_2_ is toxic for plants and POD detoxifies them (Bano and Muqarab, 2017; Batool et al., 2020). Peroxidases are the most important enzyme and they participate in different physiological processes of the plant (Almagro et al., 2009). Besides ROS scavenging, peroxidase encoding genes are also involved in lignin and suberin production to link the cell wall components. POD belongs to the PR-protein9 subfamily. The highest expression of POD increases the structure barrier and stops the infection. The expression of peroxidase genes in plants was induced by biotic and abiotic stresses (Sasaki et al., 2007). Thioredoxin genes were found to be associated with ROS scavenging enzymes such as ascorbate, POD, SOD, and catalase through disulfite bridge (Dos Santos and Rey, 2006), Moreover, Glutathione *S*-transferase detoxify electrophilic compounds in the scavenging process, also interact with plant thioredoxin (Yamazaki et al., 2004). Several stress conditions accumulate high ROS levels and resulted in the increased expression of the thioredoxin gene to participate in oxidative stress-linked signaling to induce high levels of antioxidant enzymes in potato plants (Dos Santos and Rey, 2006).

#### Genes Inhibiting Insect Digestive Enzyme

Polyphenol oxidase is a plant antioxidant enzyme important for regulating insect feeding, growth, development and defense response against herbivory and its production can be associated with the expression of different proteins and enzymes that can restrict the nutrient uptake of insects and attack their digestive track, e.g., chitinase, protease inhibitors and α-amylase inhibitors (Abebe). The chitinase enzyme is very important and it inhibits insect chewing and killing by degrading the insect skeleton (Zhong et al., 2021). The highest expression of chitinase reduces the insect induced damage and survival rate. Bordoloi et al. (2021) also identified two chitinase genes *viz*. TEA028279 and TEA019397 in tea plants with high expression under stress conditions with jasmonate and salicylic acid in their promoter upstream region to degrade chitin, of insect’s exoskeleton. Similarly, defensin gene also prevents the insect chewing by inhibiting insect’s digestive enzyme such as α-amylase and protease and makes it difficult to breakdown plant starch. Defensin express continuously in leaves stomatal cells, cell wall, peripheral layer of root, seed, and flowers, which indicate that they play an important role in protecting all entry points of plants from pest and pathogens. Khairutdinov et al. (2017) reported the inhibition of α-amylase activity by *PsDef1* defensin gene in pine pest *Panolis flammea.* The inhibitory action of α-amylase by the defensin gene in *Sorghum bicolor, Vigna unguiculata*, and *Tephrosia villosa* have also been studied (Kovaleva et al., 2020). Similar to defensin, protease inhibitors and β-glucosidase are also important for inhibiting insect gut digestive enzymes like proteases to prevent its feeding. Guedes and Cutler (2014) reported the role of insecticidal protease inhibitors in tobacco, rice, oilseed rape, sugarcane, and brassica. Expressions of protease inhibitors have been detected in wounds and induce defense against plant herbivory and pathogen attack (Clemente et al., 2019). Maize, almond, and white mustard defensive β-glucosidases were shown to restrict the digestion of lepidopteran larvae (Vassão et al., 2018).

High accumulation of metabolites contributes to acclimation of maize pre-inoculated with entomopathogenic fungi in response to *O. furnacalis* stress. In our study, significant accumulation of organic acids, benzoxazinoids, phenylpropanoids and polyketides, and lipid-like molecules were observed with higher levels in consortium treatment. Benzoxazinoid is an important defensive secondary metabolite in maize defense against *O. furnacalis* (Guo et al., 2019). However, this untargeted metabolome profiling was performed for the first time in maize plants inoculated with a consortium of *B. bassiana* and *T. asperellum* in response to *O. furnacalis* attack. We found that the contents of benzoxazinoids were changed after insect attack but higher contents were seen in inoculated treatments as compared to control. Accumulation of organic compounds plays a key role in defense against biotic and abiotic stresses (Zhang et al., 2020). We found that the accumulation of metabolites belonging to benzoxazinoids, lipid-like molecules, organic compounds and phenylpropanoids can be a common mechanism in plant adaptation to different stresses. JA contents, expression levels of genes involved in JA biosynthetic pathway and JA-dependent metabolite adjustment in lipid-like molecules, which plays a defensive role, increased during *O. furnacalis* feeding in consortium inoculated plants, indicating its enhanced protective mechanism in JA-dependent maize resistance to *O. furnacalis*. Similar findings were reported by Guo et al. (2019) in *O. furnacalis* induced direct and in direct defenses. Jasmonates are responsible for regulating nearly all biosynthetic pathways that lead to metabolites (Martínez-Medina et al., 2021). According to the significant impact of *B. bassiana* and *T. asperellum* on JA, significant changes in secondary metabolites were triggered. Similarly, previous research also reported a strong impact on plant metabolites, primary metabolism, and changes in defensive compounds (Martínez-Medina et al., 2021).

Among consortium of biocontrol agents used against pest attack, one acts as a stress inducer and the other acts as a control agent. The combined application of BCAs by different mechanisms effectively controlled the incidence of *Duponchelia fovealis* in strawberry plants (Araujo et al., 2020).

## Conclusion

By comprehensive physiological, biochemical, transcriptomic, metabolomic, and phytohormone analysis in maize inoculated with *B. bassiana* and *T. asperellum*, applied singly and in the consortium, we revealed enhanced maize defenses against *O. furnacalis herbivory.* Our study indicates that consortium of *B. bassiana* and *T. asperellum* can synergistically suppress *O. furnacalis* immune response and enhance maize immune response in the field by directly inducing enhanced expression of defense genes and indirectly activating antioxidants and phytohormone production as compared to normal herbivory-induced defenses. They can regulate the coordinated gene network expression related to defense signaling and antioxidants response and within these networks, certain genes make a key contribution to trait variation. Higher phytohormone content diverse metabolome abundance in consortium treatments was also highlighted in our results indicating the importance of JA, SA, and ethylene in plant defense. The correlation between the expression of these genes and enzymatic (SOD, POD, PPO, and protease) and non-enzymatic (proline) antioxidants, phytohormone synthesis, and metabolite adjustments were identified the first time in *B. bassiana* OFDH1-5, *T. asperellum* GDFS1009 consortium inoculated maize against *O. furnacalis* attack. The advantages of fungal BCAs are economic mass production, easy-to-use, sustainable control efficacy, and environmental safety suggest a bright future of mycoinsecticides in the world. Moreover, this study opens up new insight for future studies to focus on how certain changes in the gene networks and metabolites through biocontrol agents can affect above and below ground maize defense and other pathways. On the basis of current results, we believe that defense against ACB attack in maize is not a single gene-controlled process. Multiple genes are involved at the same time. Future research must focus on metabolic-gene clusters.

## Data Availability Statement

The datasets presented in this study is submitted in (https://www.ncbi.nlm.nih.gov/bioproject) repository, BioProject: PRJNA797234.

## Author Contributions

ZW and JC contributed to conceptualization and supervision. RB contributed to methodology, formal analysis, data curation, visualization, investigation, preparation, and writing of the original draft. MU contributed to software, formal analysis, review, and editing. YW provided resources. KH, MS, TZ, and SB were involved in the review and editing. All authors contributed to the article and approved the submitted version.

## Conflict of Interest

The authors declare that the research was conducted in the absence of any commercial or financial relationships that could be construed as a potential conflict of interest.

## Publisher’s Note

All claims expressed in this article are solely those of the authors and do not necessarily represent those of their affiliated organizations, or those of the publisher, the editors and the reviewers. Any product that may be evaluated in this article, or claim that may be made by its manufacturer, is not guaranteed or endorsed by the publisher.
