# Supplementary material for: Myco-Synergism Boosts Herbivory-Induced Maize Defense by Triggering Antioxidants and Phytohormone Signaling
Source: Front Plant Sci. 2022 Feb 17;13:790504. doi: 10.3389/fpls.2022.790504 (PMC8892192; doi:10.3389/fpls.2022.790504)
Supplement: Supplementary file 1 [file Data_Sheet_1.docx]

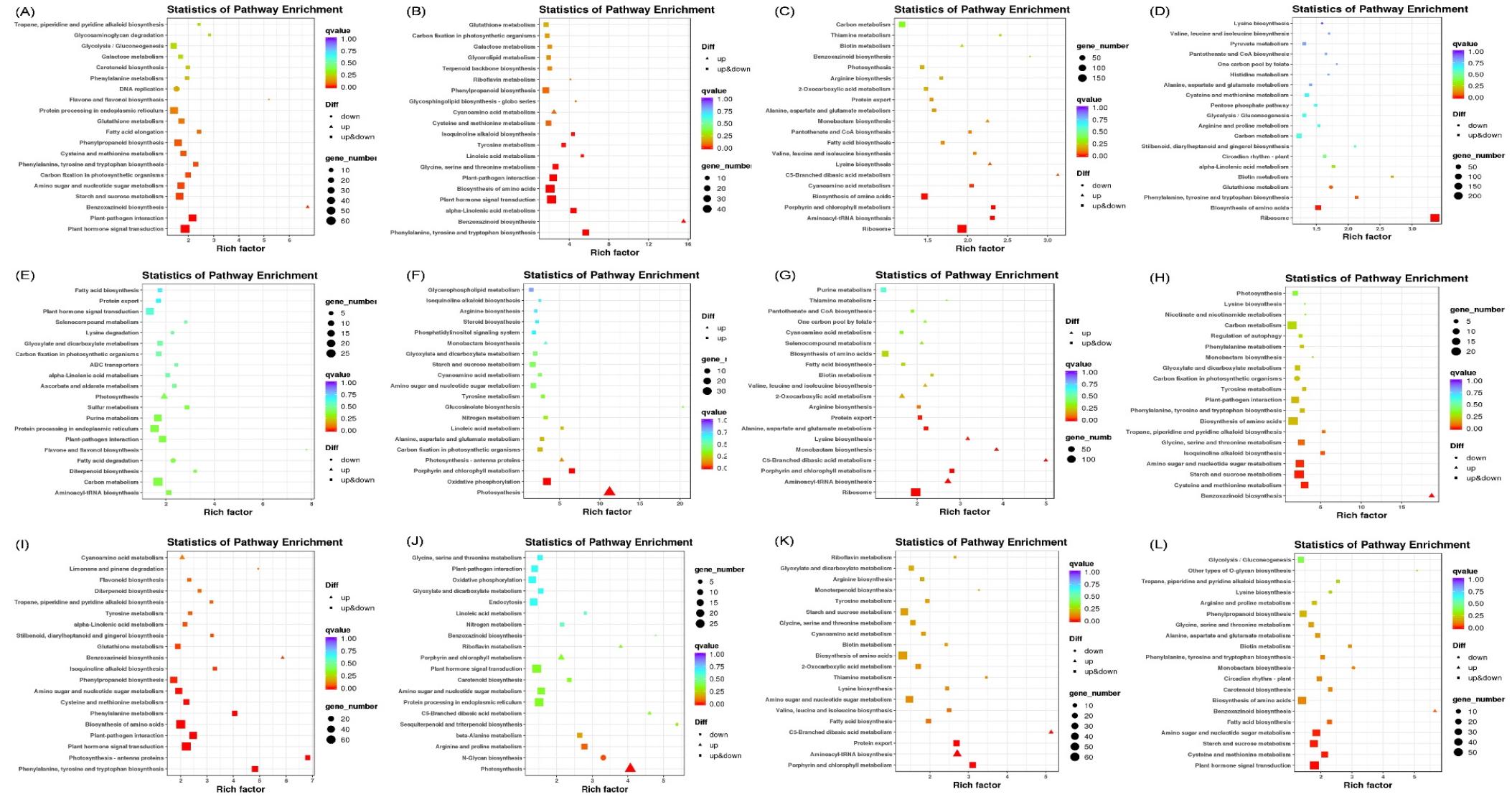


**Supplementary Figure 1**. KEGG pathway enrichment analysis of DEG's in single and consortium of B. bassiana OFDH1-5 and T. asperellum GDFS1009 inoculated maize leaves induces by O. furnacalis feeding for **(A)** IC vs BB-1(12-h): **(B)** IC vs BB-1 (24-h); **(C)** IC vs BB-1 (48-h): **(D)** IC vs BB-1 (72-h): **(E)** IC vs TH-1 (12-h); **(F)** IC vs TH-1 (24-h); **(G)** IC vs TH-1 (48-h); **(H)** IC vs TH-1 (72-h); **(I)** IC vs BT-1 (12-h); **(J)** IC vs BT-1 (24-h); **(K)** IC vs BT-1 (48-h); **(L)** IC vs BT-1 (72-h).

***
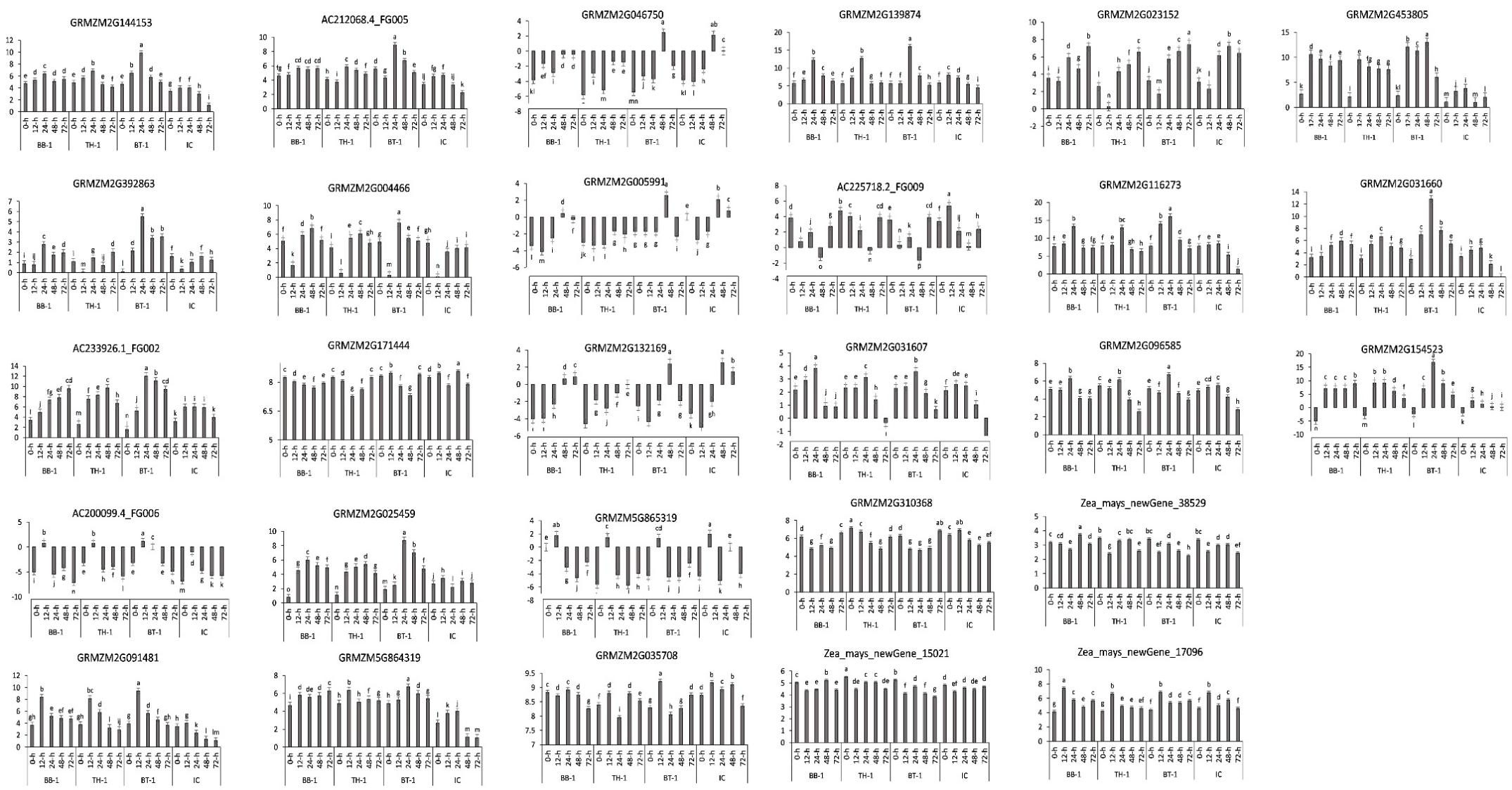
***

**Supplementary Figure 2.** qRT-PCR validation of defense related hub genes identified in single and consortium of B. bassiana OFDH1-5 and T. asperellum GDFS1009 inoculated maize leaves in response to O. furnacalis feeding.

**Supplementary Table 1.** Joint action of B. bassiana and T. asperellum against O. furnacalis.

| **Combination** | **Actual Mortality (%)** | | **Theoretical Mortality (%)** | | **Poison ratio** |
| --- | --- | --- | --- | --- | --- |
| *B. bassiana*  +  *T. asperellum* | 98.33 ± 0.073 | 60.833 ± 0.021 | | 1.6164 ± 0.027 | |

**Note:** Data is represented as mean ± standard deviation. Poison ratio>1.0 is defined as synergistic effect, =1.0 as additive effect and <1.0 as antagonistic effect.

**Supplementary Table 2.** Summary of RNA Sequencing data using maize genome as a reference.

| **Samples** | | **Clean reads** | | **Total mapped** | **GC (%)** | | **Q30 (%)** | |
| --- | --- | --- | --- | --- | --- | --- | --- | --- |
| BB-1 (0-h) | 22,283,723 | | 39,414,143 (88.44%) | | | 58.62% | | 94.88% |
| BB-1 (0-h) | 23,408,010 | | 37,907,906 (80.97%) | | | 56.82% | | 94.87% |
| BB-1 (0-h) | 24,014,097 | | 38,931,838 (81.06%) | | | 57.09% | | 94.12% |
| BB-1 (12-h) | 27,177,546 | | 47,640,354 (87.65%) | | | 56.03% | | 94.19% |
| BB-1 (12-h) | 25,708,135 | | 41,291,840 (80.31%) | | | 54.59% | | 93.34% |
| BB-1 (12-h) | 26,730,345 | | 42,579,888 (79.65%) | | | 55.61% | | 93.74% |
| BB-1 (24-h) | 26,388,444 | | 44,442,297 (84.21%) | | | 57.10% | | 93.86% |
| BB-1 (24-h) | 25,023,903 | | 41,514,800 (82.95%) | | | 57.74% | | 94.52% |
| BB-1 (24-h) | 22,483,913 | | 36,597,189 (81.39%) | | | 57.00% | | 94.10% |
| BB-1 (48-h) | 30,902,097 | | 49,728,155 (80.46%) | | | 55.69% | | 94.13% |
| BB-1 (48-h) | 30,521,204 | | 51,994,934 (85.18%) | | | 56.50% | | 93.55% |
| BB-1 (48-h) | 30,015,087 | | 52,071,300 (86.74%) | | | 56.97% | | 94.03% |
| BB-1 (72-h) | 25,546,644 | | 45,439,006 (88.93%) | | | 58.75% | | 94.77% |
| BB-1 (72-h) | 26,712,167 | | 47,437,047 (88.79%) | | | 57.38% | | 94.54% |
| BB-1 (72-h) | 20,198,165 | | 35,094,827 (86.88%) | | | 56.21% | | 94.45% |
| TH-1 (0-h) | 31,929,172 | | 56,755,341 (88.88%) | | | 57.81% | | 95.08% |
| TH-1 (0-h) | 34,299,917 | | 57,256,166 (83.46%) | | | 57.34% | | 94.63% |
| TH-1 (0-h) | 21,872,685 | | 36,034,187 (82.37%) | | | 58.17% | | 94.43% |
| TH-1 (12-h) | 22,917,231 | | 40,323,983 (87.98%) | | | 56.58% | | 94.10% |
| TH-1 (12-h) | 19,705,504 | | 31,966,941 (81.11%) | | | 55.51% | | 94.10% |
| TH-1 (12-h) | 22,384,876 | | 35,725,396 (79.80%) | | | 56.11% | | 94.76% |
| TH-1 (24-h) | 32,438,854 | | 54,228,678 (83.59%) | | | 55.43% | | 94.16% |
| TH-1 (24-h) | 20,590,128 | | 35,613,164 (86.48%) | | | 55.95% | | 94.76% |
| TH-1 (24-h) | 22,433,734 | | 39,057,063 (87.05%) | | | 57.03% | | 94.82% |
| TH-1 (48-h) | 33,075,273 | | 56,534,351 (85.46%) | | | 57.73% | | 94.58% |
| TH-1 (48-h) | 30,114,584 | | 52,358,676 (86.93%) | | | 57.52% | | 95.00% |
| TH-1 (48-h) | 23,387,389 | | 39,117,367 (83.63%) | | | 56.38% | | 94.63% |
| TH-1 (72-h) | 19,420,613 | | 34,349,515 (88.44%) | | | 57.92% | | 94.16% |
| TH-1 (72-h) | 28,991,246 | | 51,815,837 (89.36%) | | | 58.43% | | 94.78% |
| TH-1 (72-h) | 30,267,602 | | 53,998,433 (89.20%) | | | 58.56% | | 94.77% |
| BT-1 (0-h) | 34,215,075 | | 60,578,118 (88.53%) | | | 57.58% | | 94.39% |
| BT-1 (0-h) | 41,341,061 | | 70,390,447 (85.13%) | | | 56.88% | | 94.45% |
| BT-1 (0-h) | 25,331,944 | | 43,032,310 (84.94%) | | | 57.21% | | 94.60% |
| BT-1 (12-h) | 40,453,354 | | 70,344,342 (86.95%) | | | 55.43% | | 94.19% |
| BT-1 (12-h) | 21,546,502 | | 34,699,194 (80.52%) | | | 55.06% | | 93.93% |
| BT-1 (12-h) | 23,126,299 | | 38,656,482 (83.58%) | | | 56.26% | | 94.56% |
| BT-1 (24-h) | 30,512,961 | | 53,607,956 (87.84%) | | | 57.30% | | 95.00% |
| **Table 1**. Cont. | | | | | | | | |
| **Samples** | **Clean reads** | | **Total mapped** | | | **GC (%)** | | **Q30 (%)** |
| BT-1 (24-h) | 32,925,335 | | 55,580,175 (84.40%) | | | 55.97% | | 94.19% |
| BT-1 (24-h) | 21,170,137 | | 35,584,095 (84.04%) | | | 52.26% | | 94.47% |
| BT-1 (48-h) | 22,396,721 | | 38,368,073 (85.66%) | | | 57.77% | | 94.88% |
| BT-1 (48-h) | 20,626,157 | | 33,979,686 (82.37%) | | | 56.43% | | 94.39% |
| BT-1 (48-h) | 28,524,548 | | 48,769,592 (85.49%) | | | 56.18% | | 94.87% |
| BT-1 (72-h) | 33,143,646 | | 58,017,994 (87.53%) | | | 57.08% | | 94.79% |
| BT-1 (72-h) | 22,329,202 | | 39,692,265 (88.88%) | | | 57.86% | | 94.56% |
| BT-1 (72-h) | 21,622,838 | | 38,228,094 (88.40%) | | | 57.65% | | 93.81% |
| IC (0-h) | 29,496,536 | | 52,213,877 (88.51%) | | | 57.79% | | 94.38% |
| IC (0-h) | 23,218,797 | | 38,029,188 (81.89%) | | | 57.37% | | 93.24% |
| IC (0-h) | 24,216,752 | | 42,138,321 (87.00%) | | | 59.04% | | 93.90% |
| IC (12-h) | 32,200,587 | | 56,011,097 (86.97%) | | | 55.40% | | 94.70% |
| IC (12-h) | 27,775,997 | | 44,341,697 (79.82%) | | | 55.02% | | 93.94% |
| IC (12-h) | 24,893,557 | | 39,398,016 (79.13%) | | | 55.02% | | 93.84% |
| IC (24-h) | 22,641,263 | | 38,776,826 (85.63%) | | | 57.15% | | 94.14% |
| IC (24-h) | 24,895,976 | | 40,978,118 (82.30%) | | | 57.49% | | 94.32% |
| IC (24-h) | 32,641,676 | | 54,641,912 (83.70%) | | | 57.09% | | 94.48% |
| IC (48-h) | 22,676,050 | | 39,403,692 (86.88%) | | | 57.22% | | 93.98% |
| IC (48-h) | 29,791,708 | | 52,045,226 (87.35%) | | | 57.34% | | 94.30% |
| IC (48-h) | 20,601,334 | | 36,064,237 (87.53%) | | | 59.21% | | 95.08% |
| IC (72-h) | 21,927,849 | | 38,967,317 (88.85%) | | | 57.27% | | 94.72% |
| IC (72-h) | 29,009,872 | | 51,569,914 (88.88%) | | | 58.33% | | 94.49% |
| IC (72-h) | 30,399,871 | | 53,526,452 (88.04%) | | | 56.65% | | 94.23% |

**Supplementary Table 3.** All up- and down-regulated differentially expressed genes (DEG) in single and consortium of B. bassiana OFDH1-5 and T. asperellum GDFS1009 inoculated maize in response to O. furnacalis attack.

| **DEG Set** | **Total** | **Up regulated** | **Downregulated** |
| --- | --- | --- | --- |
| IC (12-h) VS BB-1 (12-h) | 3,477 | 1,705 | 1,772 |
| IC (12-h) VS TH-1 (12-h) | 1,597 | 632 | 965 |
| IC (12-h) VS BT-1 (12-h) | 3,074 | 1,723 | 1,351 |
| IC (24-h) vs BB-1 (24-h) | 2,105 | 1,152 | 953 |
| IC (24-h) vs TH-1 (24-h) | 886 | 616 | 270 |
| IC (24-h) vs BT-1 (24-h) | 2,144 | 1,209 | 935 |
| IC (48-h) vs BB-1 (48-h) | 5,536 | 3,175 | 2,361 |
| IC (48-h) vs TH-1 (48-h) | 4,106 | 2,595 | 1,511 |
| IC (48-h) vs BT-1 (48-h) | 4,205 | 2,421 | 1,784 |
| IC (72-h) vs BB-1 (72-h) | 3,255 | 1,273 | 1,982 |
| IC (72-h) vs TH-1 (72-h) | 1,230 | 543 | 687 |
| IC (72-h) vs BT-1 (72-h) | 3,105 | 1,226 | 1,879 |

**Supplementary Table 4.** Primers used for qRT-PCR.

| **Gene ID** | **Forward Primer** (5’-3’) | **Reverse Primer** (5’-3’) |  |
| --- | --- | --- | --- |
| Actin | TACCATGTTCCCTGGGATTG | GTGGCGCAATCACTTTAACC |  |
| GRMZM2G144153 | CTGCGCCATCTACAAGTTC | TCTACAGCTTTGCACCCAGA |  |
| AC212068.4_FG005 | GGATGTTCGATCACACCAGC | CGATGACCTGGTCCTCTGTT |  |
| GRMZM2G046750 | TCCAAGTCGAGTGTGTGTGA | TGCCGTTGGTGATGAAGTTG |  |
| GRMZM2G392863 | CACTGCCACTGATTCGCTC | AGGCAGACGTACGTACAACA |  |
| GRMZM2G004466 | CCACGTTTCTGTTCCTGGTG | AGCTCACAAGACCCGGTAC |  |
| GRMZM2G005991 | TGTAGGATGCATGCGTTGTG | AGAGGTGGGAGAAGAGGACT |  |
| GRMZM2G132169 | CGCAAACTCGGAAACCTTGA | GTCTTGCACAGCCTCTTCAC |  |
| GRMZM5G865319 | GGCCTCGAATTACCCTTCCT | AGAAGAAAGGATGGTCGGCA |  |
| AC233926.1_FG002 | GTCTGCAACCTGTCGAGC | TGTAGAACCTCACCCACACG |  |
| GRMZM2G171444 | CGGAGCCTGTCACTACTTCA | AGCCTCCTCAATGAACTGCT |  |
| AC200099.4_FG006 | CTCCTCTGGGCCACTTAACA | TGGAGGCTGAGGAAGAAAGG |  |
| GRMZM2G025459 | TCCTCGACCACCTGTTCATC | TCTTCCACTCACACACAGCT |  |
| GRMZM2G091481 | AACTTCATGGAGGTCGCAGA | AGGTATGGGTGGTTGTCAGG |  |
| GRMZM5G864319 | GCTTCTAAGGCTTTGTCCCG | CAGCCAGTCACCCACAAATC |  |
| GRMZM2G035708 | CACTACACCGCGAGATTTCC | TGACGCAGGTATCATCAGCT |  |
| GRMZM2G453805 | GAACAACTACAGCAGCCAGG | CTCCCGATGATCCGCTCTTA |  |
| GRMZM2G154523 | GTTCGACGTCAAGTACCTGC | TCCTTGGTGAGCATGGACAT |  |
| GRMZM2G031660 | ACCCCTTCAATTCTCCAGCA | GATCTGGTACGAGGAGGTGG |  |
| GRMZM2G139874 | CGCAGAGCTTCGAGTACAAC | CTGAGCCATCACCTTCTTGC |  |
| GRMZM2G023152 | CAGCTTCATCATGGACGTCG | TCACCGGGGACATACTTCAC |  |
| AC225718.2_FG009 | CTCCGCTCCCTCTACAACC | TGTGCATCTTCTCCTCCTCC |  |
| GRMZM2G116273 | CAGAGCTGTTGAGGGAAGGA | CACAACTTTCTGGTCGGTGG |  |
| GRMZM2G031607 | CCAGAGATGTCGTCGGATGA | TAGCTCAAATCAGAACGCGC |  |
| GRMZM2G096585 | AGAAGATTGAGGCTGCTGGT | CAGAACCTTGGTGCAAAGCT |  |
| GRMZM2G310368 | CGCAATACACATACAGCCCC | CTTCCTCACGCCTCTGTAGT |  |
| Zea_mays_newGene_38529 | GACATTCAGTGGTCGATGCC | TTGTTCGTAGCTCCACCACT |  |
| Zea_mays_newGene_15021 | GAGGACTACGATGGAGAGGC | GTTGGTCCAAGGGTTGTGTC |  |
| Zea_mays_newGene_17096 | TAAATTTACGCTCCGCCTGC | TCCTTGAACCTCGCAGTCTT |  |

**Supplementary Data 1:** All genes detected in single and consortium of *B. bassiana* OFDH1-5 and *T. asperellum* GDFS1009 inoculated maize induced by *O. furnacalis* feeding with gene expression shown in FPKM in three replicates at 0-, 12-, 24-, 48- and 72-h.

**Supplementary Data 2:** Common differentially expressed genes (DEG) in single and consortium of *B. bassiana* OFDH1-5 and *T. asperellum* GDFS1009 inoculated maize induced by *O. furnacalis* feeding with gene expression shown in mean FPKM of three replicates at 0-, 12-, 24-, 48- and 72-h.

**Supplementary Data 3:** KEGG pathway enrichment analysis of genes detected in all samples

**Supplementary Data 4:** All metabolites detected in NEG (negative) and POS (positive) mode. Expression levels were shown by FPKMs. All samples harvested form maize inoculated with single and consortium of *B. bassiana* OFDH1-5 and *T. asperellum* GDFS1009 at 0, 2, 4, 12 and 24 h following *O. furnacalis* infestation.

**Supplementary Data 5:** All Class wise Differentially annotated and expressed metabolite in NEG (negative) and POS (positive) mode. The value shown is Log 10- fold change (LOG10(FC) of each metabolite detected in maize inoculated with single and consortium of *B. bassiana* OFDH1-5 and *T. asperellum* GDFS1009 at 0, 2, 4, 12 and 24 h following *O. furnacalis* infestation
